# 

**Published:** 1881-02

**Authors:** 


Vol. I. FEBRUARY, 1881. No. 2.

### THE HOMŒOPATHIC PHYSICIAN:

### A MONTHLY JOURNAL OF MEDICAL SCIENCE.

"*Magna est veritas et praevalebit.*"

E. J. LEE, M. D.
EDITOR.

